# Sudden Loss of Vision Following Cosmetic Autologous Fat Transfer

**DOI:** 10.7759/cureus.92029

**Published:** 2025-09-11

**Authors:** Zaid Alsafi, Christelle Tendo, Zakariya Jarrar, Theresa Richardson, Rahila Zakir

**Affiliations:** 1 Department of Ophthalmology, Western Eye Hospital, London, GBR

**Keywords:** autologous fat transfer, cosmetic surgery tourism, ocular globe penetration, retinal injury, vitreo-retinal surgeon, vitreous haemorrhage

## Abstract

Cosmetic tourism and facial aesthetic procedures are increasing in popularity worldwide, with patients often seeking low-cost interventions abroad. Autologous fat transfer (AFT) is commonly performed for periorbital rejuvenation and carries a risk of rare but severe ophthalmic complications. We describe a case of unilateral visual loss following periorbital AFT, in which the underlying mechanism was scleral penetration leading to vitreous haemorrhage, retinal incarceration, and extraocular muscle entrapment. Surgical management with pars plana vitrectomy restored partial vision, although motility restriction persisted. To our knowledge, this is the first reported case of retinal incarceration caused by cosmetic AFT. This case highlights the need for careful technique, thorough patient counselling, and vigilant post-operative follow-up in cosmetic tourism.

## Introduction

Medical and cosmetic tourism has expanded significantly in recent decades, now representing a global industry valued at more than $4.5 billion in 2023 [1]. Turkey has emerged as a leading hub, ranking among the top five global destinations for aesthetic surgery [1,2]. The surge in cosmetic surgery has been fuelled by increased affordability, targeted marketing, and the influence of social media [3].

First reported in 1983, autologous fat transfer (AFT) is an established technique for periorbital rejuvenation where adipose tissue is harvested and injected into targeted areas. AFT can be performed in isolation or combined with various cosmetic procedures such as blepharoplasty, improving contour deformities and promoting skin rejuvenation through growth factor and cytokine release from adipose-derived stem cells [4]. However, AFT is not without risk. Minor complications such as bruising, oedema, and irregular contours are common but are often self-limiting [4,5]. More concerning are rare but sight-threatening complications, secondary to vascular occlusion. Several treatments have been reported in the literature such as ocular massage, vasodilators to improve retinal perfusion, and reducing intra-ocular pressure. These often have little effect, yielding poor visual outcomes [5-7].

To date, however, direct mechanical injury to the globe from periorbital AFT has not been documented. We present the first reported case of retinal incarceration and inferior rectus entrapment caused by scleral penetration during cosmetic periorbital AFT.

## Case presentation

A 49-year-old man presented to a tertiary eye unit emergency department in the United Kingdom with a five-week history of vision loss in the left eye. The symptoms followed a series of cosmetic procedures performed in Turkey under general anaesthetic, including a full set of dental veneers, chin and bilateral infraorbital rim implants, and periorbital AFT. Attempts by the patient and medical team to retrieve procedure details were unsuccessful as it transpired that the clinic had closed permanently.

**Figure 1:**
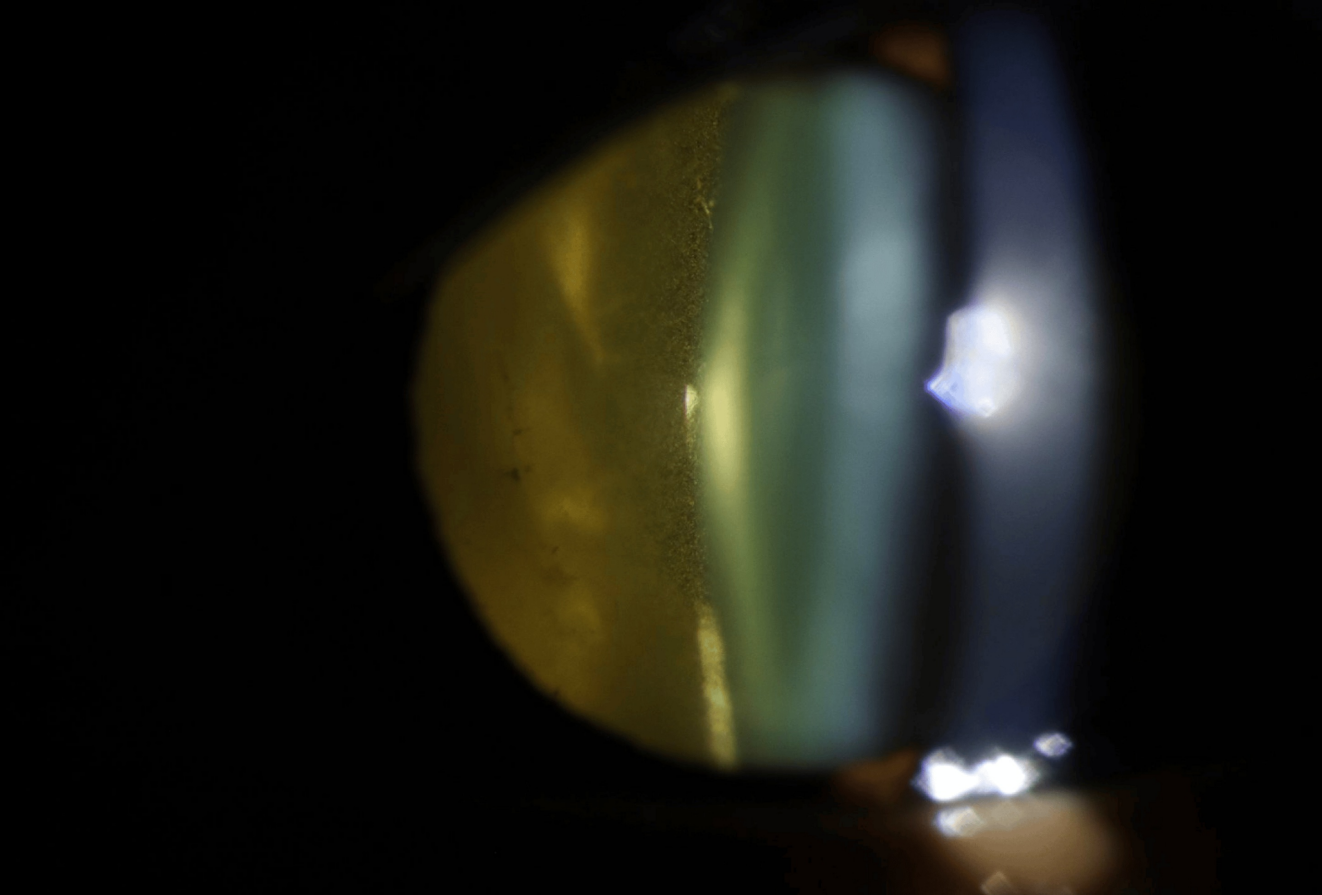
Slit-lamp photograph Magnified slit-lamp photograph of the left eye demonstrating dense white matter in the vitreous cavity precluding fundal view.

**Figure 2:**
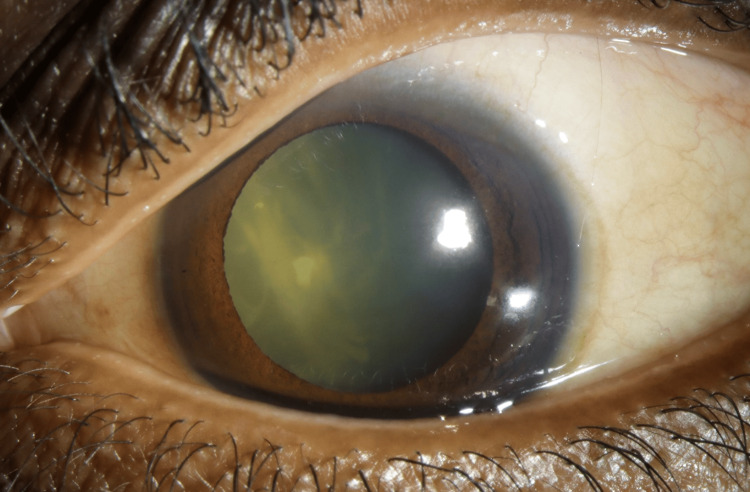
Slit-lamp photograph Slit-lamp photograph of the left eye demonstrating dense white matter in the vitreous cavity precluding fundal view.

**Figure 3:**
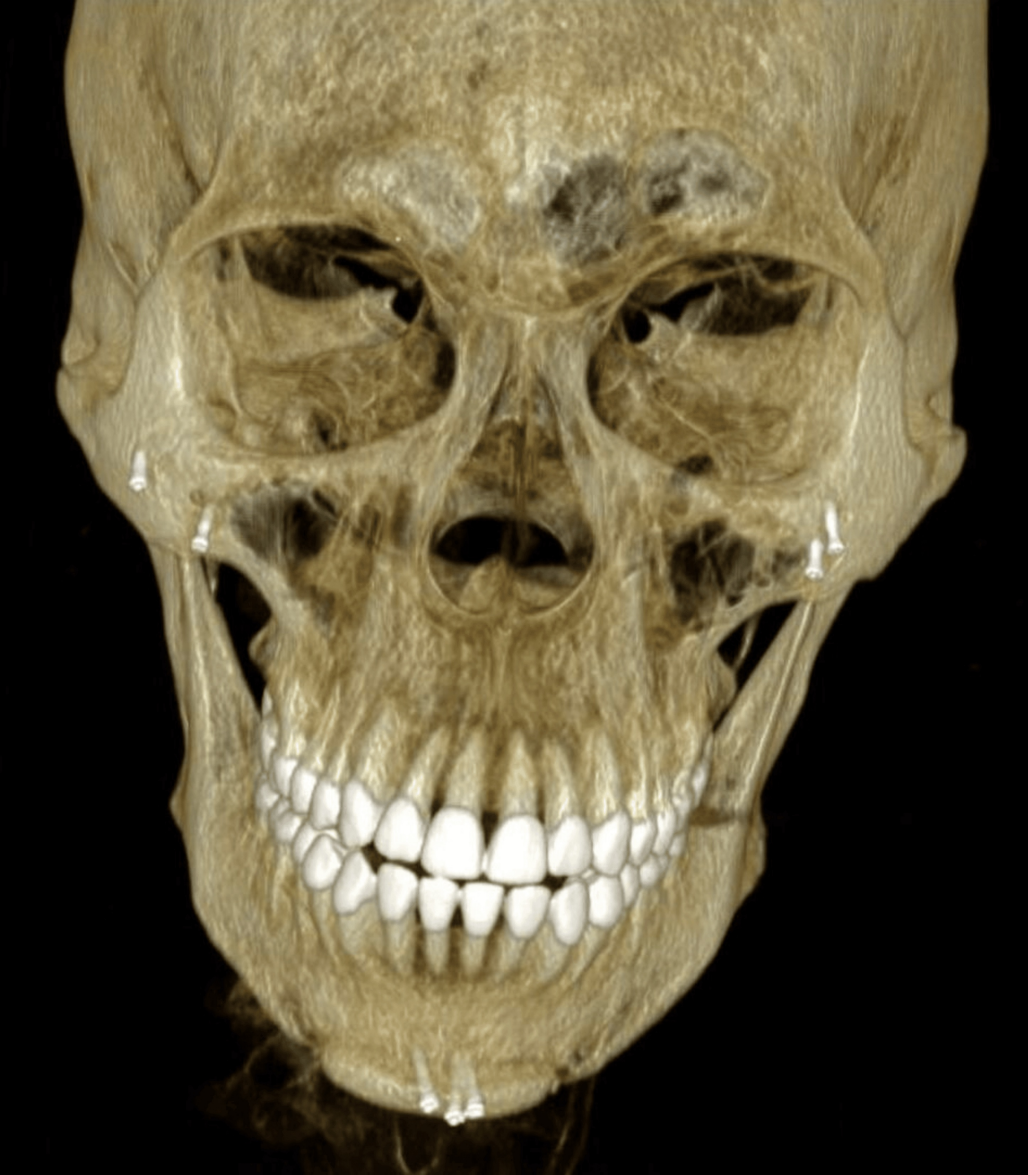
Computed tomography Three-dimensional reconstruction from a computed tomography scan of the head and facial bones demonstrating a full set of veneers as well as screws in the chin and maxillae pinning the radiolucent implants.

On examination, best-corrected visual acuity (BCVA) was −0.1 LogMAR in the right eye and perception of light in the left eye. The left eye showed dense white vitreous opacities, precluding fundal view (Figures 1, 2). B-scan ultrasonography revealed a posterior vitreous detachment with inferior tethering, a thickened hyaloid face, and a flat retina. Computed tomography (CT) of the orbit and facial bones showed bilateral infraorbital implants and a focal hyper-density in the left globe and inferior rectus muscle, without orbital inflammation or abscess (Figure 3).

**Figure 4:**
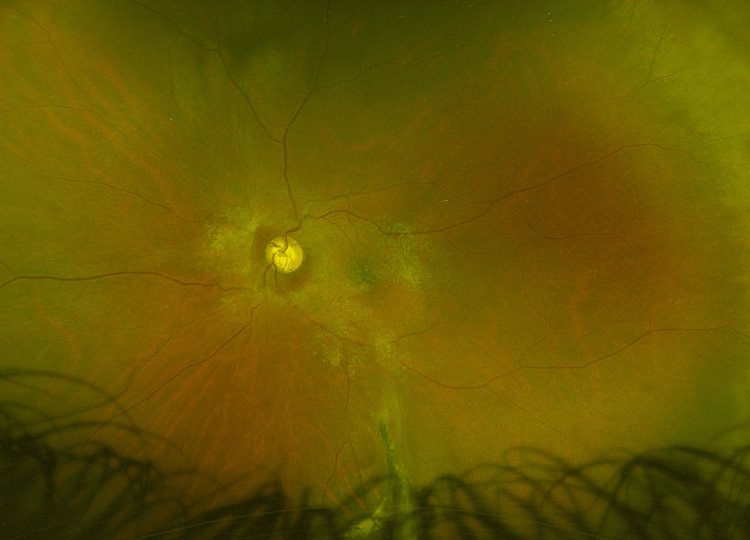
Optos ultra-widefield imaging Optos ultra-widefield imaging of the left fundus demonstrating localised tethering of the inferior sclera, consistent with prior penetrating trauma. Furthermore, an epiretinal membrane is seen, and the retina is flat.

**Figure 5:**
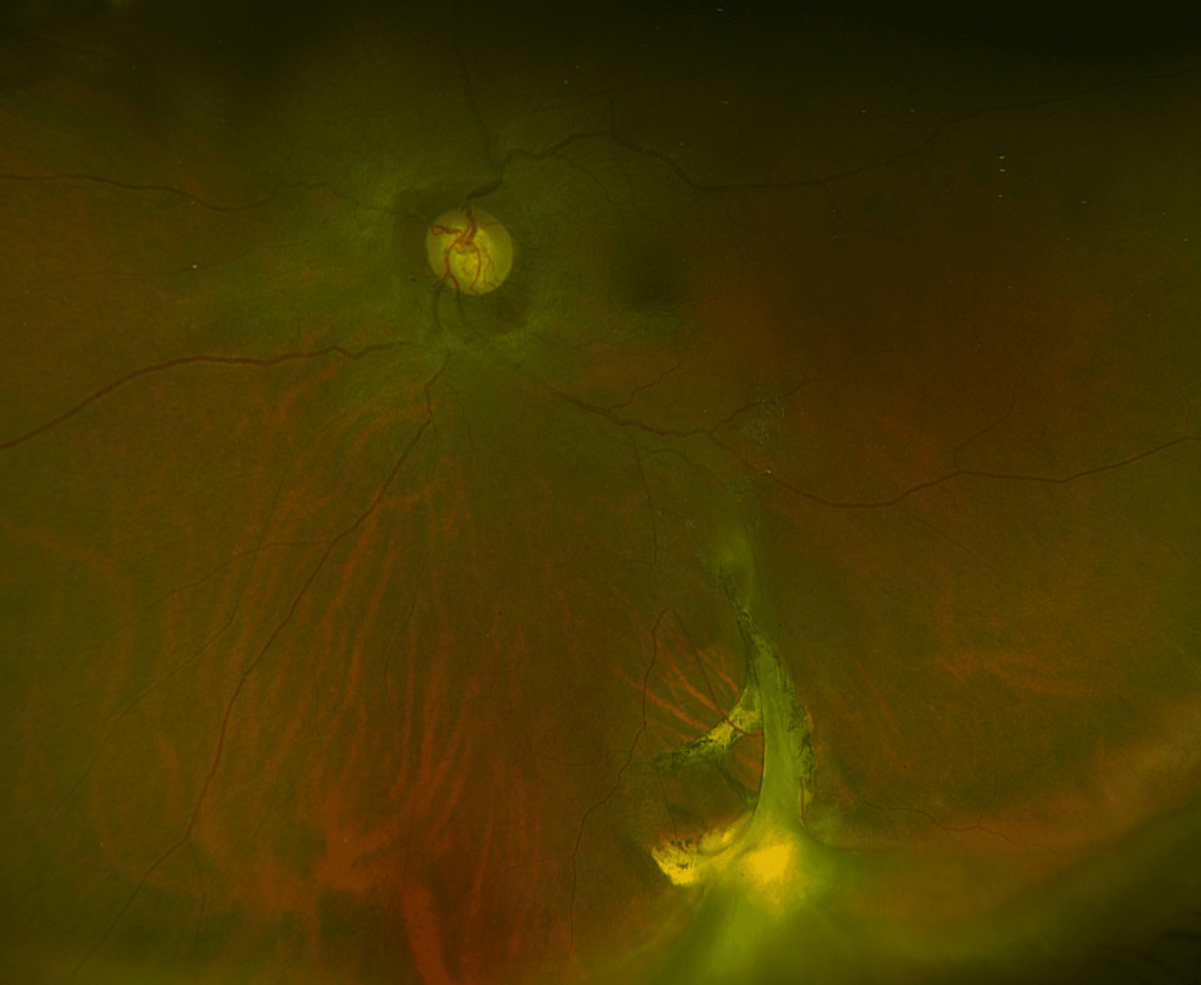
Optos ultra-widefield imaging of the inferior fundus Optos ultra-widefield imaging of the inferior aspect of the left fundus demonstrating localised tethering of the inferior sclera, consistent with prior penetrating trauma.

The patient underwent a pars plana vitrectomy. An old vitreous haemorrhage and inferior retinal incarceration associated with chorioretinal atrophy were identified. Intra-operative scleral indentation revealed localised tethering of the inferior sclera, consistent with prior penetrating trauma (Figures 4, 5).

Three days post-operatively, BCVA in the left eye improved to 0.3 LogMAR, with recovery of full colour vision. At four months, the patient remained stable, albeit with a mechanical restriction of the left inferior rectus and asymmetrical optic nerve cupping (intra-ocular pressure ranged from 12 to 16 mm Hg throughout his follow-up period). He was referred for strabismus and glaucoma evaluations.

## Discussion

AFT is widely used in cosmetic surgery, often with mild, self-limiting side effects. On the other end of the spectrum, injectable fillers have been linked to serious ophthalmic complications including, but not limited to, occlusion of the ophthalmic artery, posterior ciliary artery, central retinal artery, branch retinal artery, and posterior ischaemic optic neuropathy, which can result in permanent visual impairment and blindness [5,6]. Unlike previously documented embolic or inflammatory complications of AFT, this case represents a mechanical penetrating injury. As a result, the patient developed a vitreous haemorrhage, retinal incarceration, and extraocular muscle entrapment, expanding the spectrum of documented risks associated with AFT. Although surgical management restored partial vision, persistent motility restriction and optic nerve damage will require ongoing follow-up and intervention.

A further point of concern in this case was the patient’s inability to obtain procedural details or aftercare due to the clinic shutting down. This lack of follow-up care illustrates the dangers of cosmetic tourism, where variable standards, poor documentation, and absence of continuity of care may have a detrimental impact on patient care. In the United Kingdom, complications from cosmetic tourism are presenting more commonly to the National Health Service, resulting in significant financial implications to an already stretched system [8].

This case highlights several critical lessons. Firstly, patient education must be prioritised. Patients should be informed not only of the potential aesthetic benefits but also of the rare yet life-changing risks, such as irreversible visual impairment. Counselling allows patients to make informed decisions and encourages them to recognise complications early. Secondly, the importance of seeking care from appropriately trained clinicians in reputable clinics cannot be overstated. Ophthalmologists with expertise in orbital anatomy and cosmetic procedures are best placed to minimise risk and ensure prompt management if complications occur. Finally, structured post-operative follow-up is essential, particularly for patients traveling abroad for surgery.

## Conclusions

This case demonstrates a rare but significant complication: globe penetration with subsequent vitreous haemorrhage, retinal incarceration, and ocular muscle tethering. To our knowledge, this is the first documented report of such a complication associated with cosmetic AFT. It emphasises the need for increased awareness among ophthalmologists about the potential complications and management options following cosmetic surgery abroad. While cosmetic surgery and health tourism are not inherently problematic, patients must be adequately informed of potential risks and encouraged to seek care from reputable, qualified professionals. Improving patient education and ensuring post-procedural follow-up may help mitigate these risks.
